# Division of Os Uteri

**Published:** 1865-08

**Authors:** 


DIVISION OF OS UTERI.—The *British Medical Journal* says that Dr. Gream opposes the views of Dr. Marion Sims respecting the enlarging of the os uteri by incision. Dr. Sims, says Dr. Gream, "repudiates dilatation as dangerous in all its aspects, and declares that division of the cervix is as safe as dilatation is hazardous." Dr. Gream adds, that he has been repeatedly consulted by women who had had the os uteri divided for sterility; and never, except in one single instance, has he known a case in which pregnancy followed; and in this case the woman aborted, because the artificial opening was so great as to prevent the womb retaining its contents. He could also, he says, relate of cellulitis, pelvic abscesses, etc., following incision of the cervix. Dr. Gream considers the only proper treatment is slow and carefully managed dilatation, in properly selected cases.

